# Systemic Administration of Mesenchymal Stem Cells Increases Neuron Survival after Global Cerebral Ischemia In Vivo (2VO)

**DOI:** 10.1155/2010/534925

**Published:** 2010-12-19

**Authors:** Luisa Perasso, Carla Emilia Cogo, Debora Giunti, Carlo Gandolfo, Piero Ruggeri, Antonio Uccelli, Maurizio Balestrino

**Affiliations:** ^1^Department of Neuroscience, Ophthalmology and Genetics, University of Genova, Via De Toni 5, 16132 Genova, Italy; ^2^Department of Experimental Medicine, University of Genova, Viale Benedetto XV 3, 16132 Genova, Italy; ^3^Advanced Biotechnology Center (ABC), Largo Rosanna Benzi 1, 16132 Genova, Italy; ^4^Centre of Excellence for Biomedical Research, University of Genova, Viale Benedetto XV 7, 16132 Genova, Italy

## Abstract

Although many studies have shown that administration of stem cells after focal cerebral ischemia improves brain damage, very little data are available concerning the damage induced by global cerebral ischemia. The latter causes neuronal death in selectively vulnerable areas, including the hippocampal CA1 region. We tested the hypothesis that intravenous infusion of bone marrowderived stromal cells (mesenchimal stem cells, MSC) reduce brain damage after transient global ischemia. In adult male Sprague-Dawley rats transient global ischemia was induced using bilateral common carotid artery occlusion for 20 min in addition to controlled hypotension. Five days after, the animals were anaesthetized with urethane and the brain was fixed, sectioned and stained with hematoxylin-eosin to investigate histological damage. MSC did not fully protect against ischemic damage, as the number of viable neurons in this group was lower than in normal (sham-operated) rats. However, in MSC-treated rats the number of viable CA1 pyramidal neurons was significally higher than in rats that had been subjected to ischemia but not treated with MSC. We conclude that intravenous administration of MSC after transient global ischemia reduces hippocampal damage.

## 1. Introduction

Bone marrow (BM) contains populations of precursors that are multipotent and have the characteristics of stem cells of nonhematopoietic tissues. The precursors of nonhematopoietic tissues are referred to as bone marrow stromal cells (BMSCs) or mesenchymal stem cells (MSCs). They have attracted interest because of their capacity for self-renewal in a number of nonhematopoietic tissues and their multipotentiality for differentiation. They are able to cross the blood-brain barrier, to migrate throughout forebrain and cerebellum, and to differentiate to some extent into astrocytes and neurons. Despite their transdifferentiation potential, recent data have shown that MSCs display a significant capacity of decreasing inflammation, modulating immune responses, and protecting tissues from injuries, mostly through bystander paracrine mechanisms [1]. Cellular therapy of brain injury, including stroke and anoxic damage, stemmed from the assumption that stem cells differentiate and replace dead cells [2]. However, the usefulness of these cells in rebuilding neural networks is controversial [3], and several studies have now provided significant evidence that other mechanisms are likely to play a major role in protection and neural repair. These include induction of neurogenesis [4] and oligodendrogenesis [5], production of trophic factors [6], and protection from apoptosis [7] and from oxidative stress [8] possibly exerting an anti-inflammatory effect on cells of the innate immunity such as microglia [9] and macrophages [10]. Regardless of the mechanisms of tissue protection, several data exist concerning the effects of stem cells in the experimental therapy of focal cerebral ischemia [6, 11–14], but little research has been done in global cerebral ischemia, although encouraging data exist for this model, too [15]. In the present study, we tested the hypothesis that MSCs administered intravenously reduce histological damage after global cerebral ischemia in rats. Our hypothesis in using these cells for the treatment of global cerebral ischemia was that after crossing the blood-brain barrier MSCs preferentially reach the damaged areas in the brain [15, 16] and are able to produce cytokines and factors that can be used to reduce apoptosis and promote tissue recovery [7]. The experimental model we choose is the “two-vessel occlusion” (2VO). In this model, reversible high-grade forebrain ischemia is produced by bilateral common carotid artery occlusions combined with systemic hypotension [17]. While techniques using selected arterial occlusion better reproduce the ischemia seen in human stroke, this model of global cerebral ischemia causes a brain damage similar to that observed in patients following, for example, cardiorespiratory arrest [18]. To evaluate the damage induced by global brain ischemia, we counted the number of surviving hippocampal pyramidal cells. These cells are selectively vulnerable to global ischemic damage and can therefore gauge the effects of such a damage [19].

## 2. Materials and Methods

### 2.1. Isolation and Characterization of Mesenchymal Stem Cells

Murine bone marrow-derived MSCs were isolated from 6- to 8-week-old C57BL/6J mice (Harlan, S. Pietro al Natisone, Italy) as described elsewhere [20]. In brief, marrow cells, flushed out of tibias and femurs, were plated in 75 cm^2^ tissue culture flasks (Sarstedt, Numbrecht, Germany) at the concentration of 0,3 to 0,4 × 10^6^ cells/cm^2^ using Murine Mesencult as medium (Stem Cell Technologies, Vancouver, British Columbia, Canada). Cells were cultured in plastic plates as adherent cells and kept in a humidified 5% CO_2_ incubator at 37°C, refreshing medium every 3 days for about 6 weeks when cells reached 80% confluence. On treatment with 0.05% trypsin solution containing 0.02% EDTA (Sigma-Aldrich, St. louis, MO), marrow cells were plated in 25 cm^2^ flasks at 1.2 to 2.0 × 10^4^ cells/cm^2^ for the subsequent 4 or 5 passages. Thereafter, cells were routinely seeded at 4 to 10 × 10^3^ cells/cm^2^. Mature MSCs, obtained after four to five passages in culture, were defined by the expression on their surface of CD9, Sca-1, CD73, and CD44 antibodies and by the lack of the hematopoietic markers CD45, CD34, and CD11b.

Human bone marrow samples were obtained from healthy donors undergoing bone marrow explant for allogeneic transplantation procedures as described elsewhere [21]. Briefly, bone marrow mononuclear cells were isolated by density gradient centrifugation (1,077 g/ml; Lympholyte Cell Separation Media, Cedar Lane, Hornby, ON, Canada) and seeded at the density of 25–30 × 10^6^ cells per 75 cm^2^ tissue culture flasks in Human Mesencult as medium (Stem Cell Technologies, Vancouver, British Columbia, Canada) and incubated at 37°C and 5% CO_2_. At 80% confluence, cells were harvested with 0.05% trypsin and 0.02% EDTA and plated in 75 cm^2^ flasks at the density of 7 × 10^5^ cells. Characterization of MSCs in culture was achieved by flow cytometry. Typical CD34−, CD45−, CD14−, CD73+, CD44+, and CD105+ cells were usually obtained after three passages in culture.

### 2.2. Induction of Global Forebrain Ischemia

Adult male Sprague-Dawley rats weighing 270–300 g were obtained from Harlan Italy San Pietro al Natisone, Udine, Italy. The animals were fasted during the night preceding the operation with free access to water. Anesthesia was induced with isoflurane (5% induction, 1-2% maintenance) in a mixture of N_2_O and O_2_ (50 : 50) by a face mask. The surgical operations were made on a heated pad. Transient global ischemia was induced by bilateral occlusions of the common carotid arteries for 20 minutes. Ischemia duration was calculated as previously reported [22], leading to a reproducible neuronal damage in the CA1 region of the hippocampus, cortical layers 3, 5, and 6 and the basal ganglia. Briefly, both femoral arteries and right femoral vein were exposed and catheterized with Teflon catheters. The left femoral artery was connected to a pressure transducer (Spectramed Statham P23XL, Viggo-Spectramed, Oxnard, CA, USA) providing a recording of arterial pressure through a Grass preamplifier, model 7P14A (Grass Instruments, Quincy, MA, USA), for continuous recording of arterial pressure. The reversible forebrain ischemia was induced by combination of bilateral carotid artery clamping and reduction of mean arterial blood pressure. The carotid arteries were occluded with stainless microvascular clamps when blood pressure had reached 30 mmHg and then maintained at 30 ± 1 mmHg by blood withdrawal or reinfusion by the cannula in the right femoral artery. Ischemia was terminated after 20 minutes by removal of carotid clamps and by reinfusion of plasma volume expander (untreated ischemic, *N* = 4) or of 1mL of either human or murine MSC bolus (3 · 10^6^ MSC/ml saline) (treated ischemic, *N* = 3) by the cannula in the left femoral vein. 3 rats represented the sham-operated group; they were subjected to the surgical procedures except the ischemia and the pressure control. After the procedure, each rat recovered in a heated chamber for 2 hours then was returned to a light- and temperature-controlled facility.

### 2.3. Histology

Five days after ischemia, animals were anaesthetized with urethane (150 mg/100 g bw ip) and transcardially perfused with saline followed by fixation with ice-cold 4% paraformaldehyde in phosphate buffer. The brains were postfixed in ice-cold 4% paraformaldehyde for one hour then cryoprotected in 30% sucrose. Coronal cryostat sections (8 *μ*m) were mounted onto poly-L-lysine-coated slides. Sections were stained with hematoxylin eosin.

### 2.4. Quantification of the Ischemic Damage

Hematoxylin-eosin-stained sections were used to assess the extent of ischemic damage. 20 coronal sections were taken between 3.8 and 3.96 mm posterior to the bregma. The neuronal count was made under a light microscope at 20x magnification in 12 randomly selected hippocampal sections of 1,7 · 10^4^ *μ*m^2^ in the CA1 region. The number of neurons exhibiting ischemic cell change and morphologically normal neurons was counted in each selected hippocampal section. Ischemic neurons were defined by an intense, darkly stained pyknotic nucleus, surrounded by eosinophilic cytoplasm. Statistical analysis was carried out with analysis of variance (ANOVA) followed by Bonferroni's Multiple Comparison Test. All computations were done using the program GraphPad Prism, version 4.03 for Windows, by GraphPad Software, San Diego California (USA).

## 3. Results

**Figure 1:**
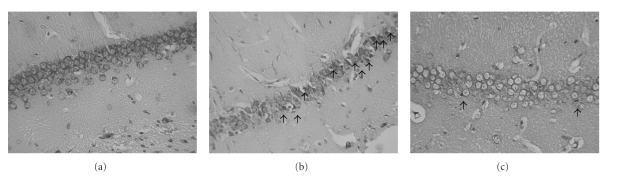
Microphotographs (20x) of pyramidal neurons in the CA1 region of the hippocampus. Sections from (a) sham-operated rat, (b) ischemic untreated, and (c) ischemic MSC-treated rat. Shrunken neurons are indicated by arrows. Images were obtained 5 days after ischemia.

**Figure 2:**
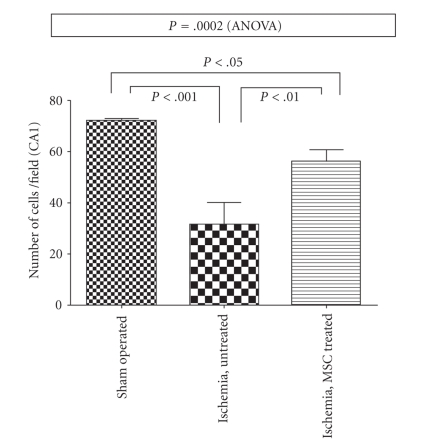
Number of vital neurons after ischemia in the various groups. Bars show mean and standard deviation. Probability values are for one-way analysis of variance (ANOVA) and for post hoc Bonferroni's Multiple Comparison Test.

Data were obtained from 10 rats, treated as follows: 3 sham-operated, 4 ischemic untreated, 2 ischemic treated with human MSCs, and 1 ischemic treated with mouse MSCs. Results are summarized in Table 1. Since all MSC-treated rats showed superimposable results (see Table 1), their data were pooled for further statistical analysis.  Figure 1 shows representative examples of microphotographs of pyramidal neurons of CA1 hippocampal region of sham-operated (a), ischemic-untreated (b), and ischemic MSC-treated rats (c). Average neuronal count (cells/field) in the CA1 hippocampal region was (mean ± SD) 72.05 ± 0.88 for sham-operated rats (*N* = 3), 32 ± 9 for untreated ischemic rats (*N* = 4), and 56 ± 5 for MSC-treated ischemic rats (*N* = 3).  Figure 2 graphically reports these values and the corresponding statistical analysis. Analysis of variance shows a statistically significant difference, and post hoc tests show that all 3 groups are statistically different from each other. As expected, ischemic-untreated rats were worse than sham-operated ones (*P* < .001). Ischemic MSC-treated rats were better than ischemic untreated, having a significantly higher number of surviving cells (*P* < .01). However, count of surviving neurons in ischemic MSC-treated rats was lower than in sham-operated rats (*P* < .05), thus showing that MSC treatment did not cause a total protection of the ischemic neurons.

## 4. Discussion

The present study investigated the therapeutic potential of MSCs in a rat model of diffuse neuronal damage induced by global ischemia. Our results suggest that intravenously injected MSCs have a positive effect on neuronal survival in this type of neuronal damage. In fact, we showed that the number of surviving neurons in the CA1 region of the hippocampus of ischemic, MSC-treated rats is significantly higher than that of ischemic, untreated ones. As a word of caution, MSC-treated rats were still worse than fully normal (sham-operated) animals, thus showing that this therapy, albeit effective, does not cause a full protection of the tissue. As for the mechanisms that improve ischemic damage upon MSCs injection after global ischemia, our data do not directly address the mechanisms responsible for the therapeutic effects of intravenous administration of bone marrow cells on cerebral infarction. One possibility may be that the MSC integrate into the tissue and replace damaged cells; however, the short time elapsed from ischemia to assessment (5 days) makes it, in our opinion, very unlikely. Despite the fact that a longer observation could have allowed to detect any further improvement due to the possible long-term capacity of providing tissue repair in the ischemic brain, the lack of evidence of engraftment in this study and, more important, the large body of evidence arising from the current literature [1, 3] suggest that, MSC transdifferentiation into neural cells does not play a major role in brain tissue repair. Since the reconstruction of neural circuitry is not always essential for functional recovery [23], a more reasonable hypothesis is that MSC therapeutic effect is based on bystander mechanisms leading to tissue protection and repair. In particular, MSC intravenous administration has been shown to lead to production of trophic factors [6, 24], which may be the functional consequence of inflammatory, hypoxic, and oxidative stress-associated insults [25]. Moreover, MSC in vivo administration results also in the downregulation of several stress-associated molecules involved in oxidative stress detoxification [26]. Finally, MSCs rescue also ischemic neurons from apoptosis [7]. All these features are supported by the global gene expression profile of therapeutic MSCs, which is enriched by anti-inflammatory, antiapoptotic, and trophic genes [27]. The results provided in this study confirm that MSCs are endowed with a remarkable therapeutic plasticity potentially useful for a vast range of diseases sustained by multiple pathogenic mechanisms. Due to their known safety in humans, further studies could expand pioneer experience with MSCs in stroke [28].

## Figures and Tables

**Table 1:** Number of cells/field in the various experiments. Same data as Figure 2, however, note that in Figure 2 the 2 MSC groups (human and mouse) were pooled. See text for more information.

Sham operated	Ischemia, untreated	Ischemia, human MSCs	Ischemia, mouse MSCs
72	28	59	59
71	27	51	
73	27		
	44		
